# Does Non-Central Nervous System Tuberculosis Increase the Risk of Ischemic Stroke? A Population-Based Propensity Score-Matched Follow-Up Study

**DOI:** 10.1371/journal.pone.0098158

**Published:** 2014-07-21

**Authors:** Chueh-Hung Wu, Li-Sheng Chen, Ming-Fang Yen, Yueh-Hsia Chiu, Ching-Yuan Fann, Hsiu-Hsi Chen, Shin-Liang Pan

**Affiliations:** 1 Department of Physical Medicine and Rehabilitation, National Taiwan University Hospital and National Taiwan University College of Medicine, Taipei, Taiwan; 2 School of Oral Hygiene, College of Oral Medicine, Taipei Medical University, Taipei, Taiwan; 3 Department and Graduate Institute of Health Care Management, Chang Gung University, Tao-Yuan, Taiwan; 4 Department of Nutrition and Health Sciences, Kainan University, Tao-Yuan, Taiwan; 5 Centre of Biostatistics Consultation, College of Public Health, National Taiwan University, Taipei, Taiwan; 6 Division of Biostatistics, Graduate Institute of Epidemiology, College of Public Health, National Taiwan University, Taipei, Taiwan; Cardiff University, United Kingdom

## Abstract

**Background:**

Previous studies on the association between tuberculosis and the risk of developing ischemic stroke have generated inconsistent results. We therefore performed a population-based, propensity score-matched longitudinal follow-up study to investigate whether contracting non-central nervous system (CNS) tuberculosis leads to an increased risk of ischemic stroke.

**Methods:**

We used a logistic regression model that includes age, sex, pre-existing comorbidities and socioeconomic status as covariates to compute the propensity score. A total of 5804 persons with at least three ambulatory visits in 2001 with the principal diagnosis of non-CNS tuberculosis were enrolled in the tuberculosis group. The non-tuberculosis group consisted of 5804, propensity score-matched subjects without tuberculosis. The three-year ischemic stroke-free survival rates for these 2 groups were estimated using the Kaplan-Meier method. The stratified Cox proportional hazards regression was used to estimate the effect of tuberculosis on the occurrence of ischemic stroke.

**Results:**

During three-year follow-up, 176 subjects in the tuberculosis group (3.0%) and 207 in the non-tuberculosis group (3.6%) had ischemic stroke. The hazard ratio for developing ischemic stroke in the tuberculosis group was 0.92 compared to the non-tuberculosis group (95% confidence interval: 0.73–1.14, P = 0.4299).

**Conclusions:**

Non-CNS tuberculosis does not increase the risk of subsequent ischemic stroke.

## Introduction

Tuberculosis is an important disease that causes chronic infection worldwide [1]; the World Health Organization (WHO) estimates that there were 13.7 million prevalent cases in 2007, with 9.27 million new cases [1]. Seroepidemiological studies have suggested a positive association between chronic infections and the risk of developing atherosclerosis and stroke [2]–[7]. However, there have been few studies exploring the relationship between tuberculosis and cardiovascular risk and they led to conflicting conclusions. Giral et al. [8] performed an age- and sex-matched case control study and showed that past tuberculosis was not associated with a higher prevalence of atherosclerotic lesions in hypercholesterolemic patients. In contrast, Sheu et al. [9] performed a population-based three-year follow-up study using an insurance database and demonstrated an increased risk of ischemic stroke after contracting non-central nervous system tuberculosis. Since stroke is a highly disabling disease resulting in enormous socioeconomic burden, clinicians should be alert to the possibility of stroke when treating tuberculosis if a positive association does exist. However, in the study of Sheu et al., the tuberculosis group was significantly older and with a higher prevalence of males and a 2-fold higher prevalence of diabetes than the comparison group (diabetes prevalence 11.6% vs. 6.0%) [9]. Because aging, male sex, and diabetes are well-known risk factors for stroke [10]–[13], this raises the possibility that the observed association between tuberculosis and stroke was, at least in part, due to the substantial imbalance in the distribution of age, sex, diabetes, and vascular risk factors, rather than to tuberculosis itself. One approach to minimize such confounding effects is to match the subjects with and without tuberculosis in terms of demographic and clinical characteristics [14], [15]. Propensity score matching methods are increasingly being used in observational studies to reduce bias [16]–[19]. In the present study, we aimed to investigate whether there is an increased risk of ischemic stroke after contracting tuberculosis by performing a large-scale propensity score-matched follow-up study.

## Materials and Methods

### Data source

The data used in this study were available from the complete National Health Insurance (NHI) claim database in Taiwan for the period 2000 to 2003. The NHI program has been implemented in Taiwan since 1995, and the coverage rate was 96% of the whole population in 2000 and 97% at the end of 2003, at which time more than 21.9 million inhabitants were enrolled. It should be noted that the rationale for using the NHI database after 2000 is that, from Jan 1, 2000, according to the rules of the Bureau of NHI, the NHI claim data were all encoded using the standardized International Classification of Disease, 9^th^ Revision, Clinical Modification (ICD-9-CM). To keep individual information confidential in order to satisfy regulations on personal privacy in Taiwan, all personal identification numbers in the data were encrypted by converting the personal identification numbers into scrambled numbers before data processing. Because the database used in this study consists of de-identified secondary data released for research purposes, this principle complies with the Personal Information Protection Act in Taiwan, and this study was exempt from full review by National Taiwan University Hospital Research Ethics Committee.

### Study subjects and design

We used a cohort study design to investigate the effect of non-central nervous system (CNS) tuberculosis on the risk of developing subsequent ischemic stroke. To reduce potential confounding effects from imbalance in clinical characteristics, we used propensity score matching to create comparable cohorts between patients with and without tuberculosis [20], [21]. The study population consisted of a tuberculosis group and a non-tuberculosis group, both of which were selected from Taiwanese residents in the complete NHI claims database for 2001, in which more than 21.6 million persons were registered. The tuberculosis group consisted of subjects aged 18 years or older who had received a principal diagnosis of tuberculosis (ICD-9-CM codes 010, 011, 012, 014, 015, 016, 017, and 018), but not tuberculosis of the meninges and CNS (ICD-9-CM code 013), in ambulatory medical care visits between January 1, 2001 and December 31, 2001. To maximize case ascertainment, only patients who had at least 3 ambulatory visits with the principal diagnosis of tuberculosis and had received antituberculosis medication, such as isoniazid, rifampin, ethambutol, or pyrazinamide, in this period were initially considered for inclusion in the tuberculosis group (n = 20225). The index visit was defined as the first ambulatory visit during which a principal diagnosis of tuberculosis was made. The exclusion criteria for the tuberculosis group were: (1) a previous diagnosis of any type of tuberculosis (ICD-9-CM codes 010, 011, 012, 013, 014, 015, 016, 017, and 018) during 2000 (n = 13847) to increase the likelihood of identifying only new tuberculosis cases in 2001 and (2) a previous diagnosis of any type of stroke (ICD-9-CM codes 430 - 438) before their index ambulatory care visit (n = 1970). A total of 5846 subjects was identified in the tuberculosis group.

### Covariates and propensity score matching

The information of pre-existing comorbidities, including diabetes (ICD-9-CM code 250), hypertension (ICD-9-CM codes 401–405), hyperlipidemia (ICD-9-CM code 272), coronary heart disease (ICD-9-CM codes 410–414 and 429.2), chronic rheumatic heart disease (ICD-9-CM codes 393–398), and other types of heart disease (ICD-9-CM codes 420–429), were acquired by tracking all the ambulatory medical care and inpatient records in the NHI database in the year before the index visit. The case ascertainment for these medical comorbidities was defined from ≥1 hospital discharge or ≥2 ambulatory visits with a relevant principal or secondary diagnosis code. Previous studies have suggested that the risk of stroke may be affected by socioeconomic status such as geographical regions, levels of urbanization, and income levels [22], [23]. Therefore, these factors are also taken into account as variables in assessing the risk of stroke. The information of the geographical location of residency of each subject was obtained from the population household registry. The geographical location of residency was classified into Northern, Central, Eastern, and Southern Taiwan. In accordance with Taiwan National Health Research Institute publications [24], urbanization levels in Taiwan are classified into 7 strata, with level 1 referring to the “most urbanized” and level 7 referring to the “least urbanized” communities. However, since there were relatively small number of subjects in levels 5, 6, and 7, these 3 levels were merged into a single group and labeled as level 5. For the income level, we used the insured payroll-related amount as a proxy for income (0, NT$1 to NT$15840, NT$15841 to NT$25000, NT$25001; NT$ indicates new Taiwan dollar). Note that we selected NT$15840 as the first cutoff point of income level because this is the government-stipulated minimum wage for full-time employees in Taiwan. The use of the insured payroll-related amount as a proxy for income has been extensively used in many publications using the same insurance database [9], [25], [26]. We used these cutoff points to be in accordance with other studies sharing the same data source. Since the household registry information is not available in 42 subjects out of the 5846 subjects in the tuberculosis group, these 42 subjects were excluded from the analysis. The final tuberculosis group consisted of 5804 subjects.

The non-tuberculosis group was taken from the remaining subjects without a diagnosis of tuberculosis in the same 2001 NHI claim database. We assigned the first ambulatory medical care visit during 2001 as the index ambulatory visit. The exclusion criteria for recruiting subjects into the non-tuberculosis group were: (1) a previous diagnosis of any type of tuberculosis (ICD-9-CM codes 010, 011, 012, 013, 014, 015, 016, 017, and 018) before the index visit and (2) a previous diagnosis of any type of stroke (ICD-9-CM codes 430–438) before the index visit. The information of pre-existing co-morbidities and socioeconomic status were acquired using the same methods described above. Because the number of subjects in the NHI database is very large, we used a two-stage method to select the propensity score-matched non-tuberculosis group. For each subject in the tuberculosis group, we first randomly sampled 20 age and sex-matched non- tuberculosis subjects who met the abovementioned criteria. A total of 116080 non-tuberculosis subjects was initially sampled. In the second stage, a logistic regression model including age, sex, pre-existing co-morbidities and socioeconomic status as covariates was used to predict the probability (i.e. propensity score) of tuberculosis. An 8-to-1 greedy matching algorithm [20] was then used to identify a unique matched control from the 116080 non-tuberculosis subjects for each tuberculosis patient according to the propensity score. A total of 5804 subjects was selected in the propensity score-matched non- tuberculosis group.

### Outcome

All the medical care records for each subject in the tuberculosis and non-tuberculosis groups were tracked from their index visit for 3 years and the mortality data for the subjects who died during the follow-up were obtained from the national mortality registry. The date of the first occurrence of a principal diagnosis of ischemic stroke (ICD-9-CM codes 433–437) within the follow-up period was defined as the primary endpoint. The case ascertainment for stroke required ≥1 hospital discharge or ≥2 ambulatory medical care visits with the principal diagnosis of ischemic stroke. All subjects were followed from the index visit to the first occurrence of ischemic stroke, death, or end of follow-up.

### Statistical analysis

The Chi-square test and student's t test were used to examine differences in demographic variables, comorbid medical disorders, and propensity scores between the tuberculosis and non-tuberculosis groups. The ischemic stroke-free survival curves of the propensity-score matched tuberculosis and non-tuberculosis groups were generated using the Kaplan-Meier method and the difference in survival between these two groups was assessed using the log-rank test. Stratified Cox proportional hazard regression with patients matched on propensity score was used to estimate the effect of tuberculosis on the occurrence of ischemic stroke. We assessed the proportional hazard assumption by including time dependent terms in the Cox regression model. The analyses suggest that the proportional hazard assumption was not violated. An alpha level of 0.05 was considered statistically significant for all analyses. The analyses were performed using SAS 9.2 software (SAS Institute, Cary, NC).

## Results

The left part of Table 1 shows the demographic and clinical characteristics of the tuberculosis and non-tuberculosis groups before propensity score matching. The tuberculosis group had a higher prevalence of diabetes (P<0.0001), atrial fibrillation (P = 0.0007), chronic rheumatic heart disease (P = 0.0285) and other heart disease (P<0.0001) and a lower prevalence of hypertension (P<0.0001) and hyperlipidemia (P = 0.0345) compared to the non-tuberculosis group. There were also significant differences in the distribution of monthly income, urbanization level, and geographic region between the tuberculosis and non-tuberculosis groups. The tuberculosis groups had higher propensity score than the non-tuberculosis group (P<0.0001). There was lacking of significant difference in the prevalence of coronary heart disease (P = 0.7626) between the two groups. After propensity score matching, the matched cohorts were well-balanced in terms of all observed covariates (right part of Table 1). There was no statistically significant difference in all the baseline characteristics between the tuberculosis group and matched non-tuberculosis group.

**Table 1:** Demographic characteristics and comorbid medical disorders for the Tuberculosis (TB) and Non-TB groups before and after propensity score matching.

	Before matching			After matching		
Variables	TB group (N = 5804)	Non-TB group (N = 116080)	*p* value	TB group (N = 5804)	Non-TB group (N = 5804)	*p* value
Sex(women)	1922 (33.1)	38440 (33.1)	1.0000	1922 (33.1)	1925 (33.2)	0.9528
Age(years)	53.0±18.7	52.7±18.7	0.5086	53.0±18.7	53.2±18.9	0.6337
Diabetes(yes)	726 (12.5)	9419 (8.1)	<0.0001	726 (12.5)	751 (12.9)	0.4862
Hypertension(yes)	909 (15.7)	22952 (19.8)	<0.0001	909 (15.7)	933 (16.1)	0.5421
Hyperlipidemia(yes)	277 (4.8)	6285 (5.4)	0.0345	277 (4.8)	259 (4.5)	0.4260
Atrial fibrillation(yes)	51 (0.9)	628 (0.5)	0.0007	51 (0.9)	55 (0.9)	0.6963
Coronary heart disease	432 (7.4)	8517 (7.3)	0.7626	432 (7.4)	448 (7.7)	0.5748
Chronic rheumatic heart disease(yes)	39 (0.7)	544 (0.5)	0.0285	39 (0.7)	32 (0.6)	0.4047
Other heart disease(yes)	450 (7.8)	6522 (5.6)	<0.0001	450 (7.8)	477 (8.2)	0.3552
Monthly income			<0.0001			0.9788
NT$0	1504 (25.9)	28781 (24.8)		1504 (25.9)	1517 (26.1)	
NT$1- NT$15840	1145 (19.7)	19576 (16.9)		1145 (19.7)	1135 (19.6)	
NT$15841- NT$25000	2253 (38.9)	44042 (37.9)		2253 (38.9)	2240 (38.6)	
≥NT$25001	902 (15.5)	23681 (20.4)		902 (15.5)	912 (15.7)	
Urbanization level			<0.0001			0.7006
1 (most urbanized)	1087 (18.7)	21654 (18.7)		1087 (18.7)	1060 (18.3)	
2	604 (10.4)	13139 (11.3)		604 (10.4)	604 (10.4)	
3	1312 (22.6)	30205 (26.0)		1312 (22.6)	1363 (23.5)	
4	856 (14.7)	17875 (15.4)		856 (14.7)	879 (15.1)	
5	1945 (33.6)	33207 (28.6)		1945 (33.6)	1898 (32.7)	
Geographic region			<0.0001			0.9445
Northern	2304 (39.7)	51567 (44.5)		2304 (39.7)	2333 (40.2)	
Central	1054 (18.2)	21728 (18.7)		1054 (18.2)	1049 (18.1)	
Southern	2136 (36.8)	39374 (33.9)		2136 (36.8)	2109 (36.3)	
Eastern	310 (5.3)	3411 (2.9)		310 (5.3)	313 (5.4)	
Propensity score	0.053±0.020	0.047±0.016	<0.0001	0.053±0.020	0.053±0.020	0.9855

Data are expressed as N (%) or mean ± SD.

US $1 =  NT $34 in 2001.

**Figure 1:**
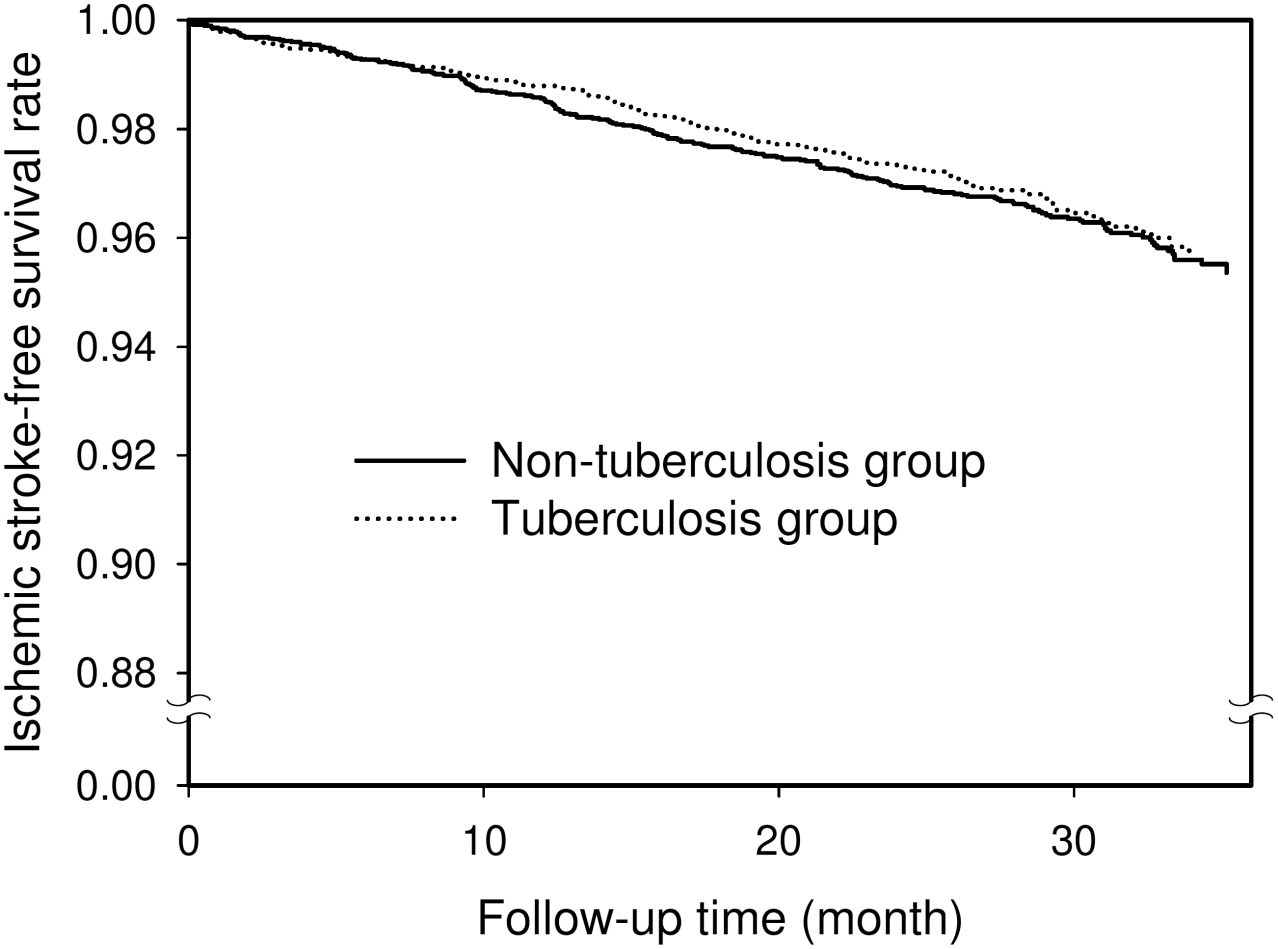
Three-year ischemic stroke-free survival rates for the tuberculosis group (dotted line) and the non-tuberculosis group (solid line).

The number of stroke events and the hazard ratios (HR) of stroke during the three-year follow-up period for the two propensity score-matched groups are presented in Table 2. Of the 5804 subjects with tuberculosis, 176 (3.0%) developed ischemic stroke compared to 207 (3.6%) of the 5804 subjects in the non-tuberculosis group. The HR of stroke for patients with tuberculosis was 0.92 (95% confidence interval [CI], 0.73–1.14, P = 0.4299). The three-year stroke-free survival rates for the two groups are shown in Figure 1. No significant difference in stroke-free survival rate was seen between the two groups (P = 0.5457).

**Table 2:** Number of ischemic stroke events, the hazard ratio of stroke for the matched Tuberculosis (TB) and Non-TB groups.

Variables	TB group (N = 5804)	Non- TB group (N = 5804)	*p* value
Ischemic stroke events, N (%)	176 (3.0)	207 (3.6)	
Hazard Ratio (95% CI)	0.92 (0.73–1.14)	1.00	0.4299

### Sensitivity analysis

In the present study, the diagnosis of tuberculosis was determined by the ICD-9-CM codes and medications from the insurance database, and may be less accurate than those obtained through a standardized procedure. Therefore, we performed a sensitivity analysis to investigate the impact of different case definitions on our results. We applied a more rigorous case definition that included only subjects that received at least 3 principal diagnosis of tuberculosis and had received least two kinds of anti-tuberculosis medications for at least 90 days. The results showed that 3671 (63.2%) out of the 5804 patients in the tuberculosis group fit this more rigorous case definition. The estimated adjusted HR of ischemic stroke for these tuberculosis patients was 0.81 (95% confidence interval [CI], 0.61 to 1.09, P = 0.1609), which is consistent with the estimates from the original analysis (Table 2, HR = 0.92, 95% CI, 0.73 to 1.14, P = 0.4299).

## Discussion

In the present large-scale population-based propensity score-matched three-year follow-up study, the occurrence of non-CNS tuberculosis was not associated with an increased risk of ischemic stroke; there was no significant difference in the three-year stroke-free survival rates between the tuberculosis and non-tuberculosis groups. These results were contrary to a modestly increased risk of ischemic stroke in three-year follow-up after the occurrence of non-CNS tuberculosis (adjusted HR = 1.52, 95% CI, 1.21–1.91, P<0.001) reported by Sheu et al. [9].

This discrepancy may be attributed to the substantial imbalance in the distribution of age, sex, and diabetes in the study of Sheu et al., which did not match these variables between the tuberculosis group and comparison group. Because tuberculosis has been associated with aging, male sex, and diabetes [27]–[29], it can be expected that, without matching, subjects with tuberculosis would be older and have a higher proportion of males and a higher prevalence of diabetes than subjects without tuberculosis, as seen in the study of Sheu et al. Since aging, male sex, and diabetes are also known risk factors for ischemic stroke [10]–[12], such imbalance in the baseline demographic and comorbidity variables may lead to a confounded association between tuberculosis and ischemic stroke. Furthermore, the potential confounding effects of these variables may not be completely removed by covariate adjustment in multiple regression analysis due to the following concerns. First, the effects of confounders may be non-linear (e.g. quadratic or exponential). However, multiple regression analysis commonly assumes these effects to be linear [16]. Second, the effects of confounders cannot be completely eliminated simply by adjusting covariates in multiple regression, unless confounders are measured without any error [16]. Matching is an alternative method for controlling confounders. However, when the number of variables for matching increases, it becomes difficult in finding close matches. Propensity scoring summarizes all measured potential confounders into a single composite score [17]. Matching on propensity score will be similar to matching on all the included covariates used for computing the propensity score. In our analysis, there was no significant difference in all demographic and comorbidities variables after matching. Therefore, potential confounding effects of these variables could be minimized by applying propensity score matching. The results showed that the occurrence of tuberculosis was not related to an increased risk of subsequent ischemic stroke. Such findings are compatible with those in the study of Giral et al. [8], which also showed that tuberculosis was not associated with carotid atherosclerosis. We therefore suggest that non-CNS tuberculosis is not linked to a higher risk of ischemic stroke.

Before matching (left part of Table 1), the tuberculosis group had higher prevalence of pre-existing diabetes but lower prevalence of hypertension compared to the non- tuberculosis group. The positive association between diabetes and tuberculosis has been well recognized [30]–[33]. To our knowledge, however, there was no literature reporting the relationship between hypertension and tuberculosis. Our study showed a significant negative association between tuberculosis and hypertension. The exact mechanism is unclear. Further studies are required to validate our findings and to elucidate the underlying mechanism.

Several strengths of this study should be addressed. First, this study used a longitudinal population-based database, which enabled us to identify all incident strokes and to evaluate the temporal relationship between tuberculosis and ischemic stroke. Second, by applying propensity-score matching, the potential confounding effects of the observed variables could be minimized. Third, the large study population was capable of providing adequate statistical power to minimize the chance of failure in detecting the association between tuberculosis and ischemic stroke. Given that our study included a total of 11608 subjects (5804 subjects with tuberculosis and 5804 subjects in the non-tuberculosis group) and a total of 383 events of ischemic stroke occurred during follow-up, our study provided 90% power to detect a hazard ratio of 0.7 for the risk of ischemic stroke between the non-tuberculosis and tuberculosis groups, with a 5% false-positive rate (2-sided tests) [34].

On the other hand, several limitations should be acknowledged. First, the diagnosis of tuberculosis, stroke, and medical comorbidities in our study was entirely determined by the ICD codes from the NHI claim data, which would be less accurate than those obtained through a standardized procedure. Moreover, due to the inherent limitation of the insurance data base, the results of smear or culture were not available. There may be concern about the diagnostic accuracy of the database. However, the Bureau of NHI has formed different audit committees that randomly sample the claim data from every hospital and review charts on a regular basis to verify the diagnostic validity and quality of care. Accordingly, the NHI claim database is an established research database and has been used in various biomedical research fields [35]. Moreover, we used case ascertainment algorithms that required at least 3 ambulatory medical care visits with a principal diagnosis code of tuberculosis and the use of antituberculosis medication to validate the diagnosis, such criteria that may be expected to provide adequate diagnostic accuracy. In Taiwan, tuberculosis is an important notifiable disease that must be reported to the health authority, which therefore reduces the possibility that subjects with tuberculosis were included in the non-tuberculosis group. Moreover, the barrier to medical access is negligible in Taiwan because the NHI system allows patients to visit any clinic or hospital freely without referral by a general practitioner, and patients pay only about $5–$15 USD at each visit. Considering the tuberculosis related symptoms (e.g. chronic cough with sputum, fever, night sweats, weight loss), and the minimal barrier to medical access in Taiwan, we believe most patients with tuberculosis would seek medical help, and the majority of tuberculosis cases are expected to have visits with physicians. Nevertheless, in the present study, since the diagnoses of tuberculosis are completely determined using the ICD codes from the insurance database, it is still possible that false negative non-tuberculosis diagnosis did exist. In addition, the diagnostic accuracy of ischemic stroke in this insurance database has been formally validated in a validation study [36]. Therefore, it can be expected that the asertainment bias of ischemic stroke occurrence is minimal. Furthermore, we performed a sensitivity analysis using different case definitions, and the results of the sensitivity analysis are consistent with the original findings, suggesting that our results are robust to different case definitions. Second, due to the inherent limitations of the NHI database, information was lacking regarding lifestyle factors, such as smoking, alcohol consumption, and obesity, which may affect the interpretation of our findings. Of these, cigarette smoking has been documented as a risk factor of tuberculosis [18], [37]. However, because smoking is expected to increase the risk of both tuberculosis and ischemic stroke, and no significant association was found between tuberculosis and ischemic stroke in our study, we believe that cigarette smoking is not likely to confound the relationship between tuberculosis and ischemic stroke in this study. Third, most inhabitants of Taiwan are of Chinese ethnicity and it is uncertain whether our findings can be generalized to other ethnic groups [38].

## Conclusions

The present large-scale population-based propensity score-matched study showed that the occurrence of non-CNS tuberculosis is not linked to an increased risk of developing ischemic stroke.
